# STAT3-mediated upregulation of LINC00520 contributed to temozolomide chemoresistance in glioblastoma by interacting with RNA-binding protein LIN28B

**DOI:** 10.1186/s12935-022-02659-y

**Published:** 2022-08-09

**Authors:** Shuai Yuan, Qi Yan, Zhi-yong Zhao, Jing-long Zhang, He Zhang, Hang Yin, Zhi Yuan

**Affiliations:** 1 Department of Neurosurgery, Lanzhou University Second Hospital, No. 82, Cuiyingmen, Gansu 730030 Lanzhou, China; 2 Department of Neurology, Lanzhou University Second Hospital, 730030 Lanzhou, Gansu China

**Keywords:** Glioblastoma (GBM), LINC00520, STAT3, LIN28B, Temozolomide (TMZ), Chemoresistance

## Abstract

A considerable number of glioblastoma (GBM) patients developed drug resistance to Temozolomide (TMZ) during chemotherapy, resulting in therapeutic failure and tumor recurrence. However, the exact mechanism of TMZ chemoresistance in GBM is still poorly clarified. As a novel identified lncRNA, LINC00520 was located on chromosome 14 and overexpressed in multiple human cancers. This study was designed and conducted to investigate the role and underlying mechanism of LINC00520 in GBM chemoresistance to TMZ. The qRT-PCR assay demonstrated that LINC00520 was significantly overexpressed in TMZ-sensitive and/or TMZ-resistant GBM cells (P < 0.001). The silencing of LINC00520 markedly reduced the cell viability, suppressed colony formation, induced cell apoptosis and G1/S phase arrest in TMZ-resistant cells (P < 0.001). In contrast, overexpression of LINC00520 conferred TMZ-resistant phenotype of GBM cells in vitro (P < 0.001). The orthotopic xenograft model was established and the results indicated that the volume of tumor xenografts in vivo was markedly inhibited by TMZ treatment after the silencing of LINC00520 (P < 0.001). Luciferase reporter assay and chromatin immunoprecipitation (ChIP) assay revealed a strong affinity of transcription factor STAT3 to the promoter regions of LINC00520, suggesting that STAT3 mediated the aberrant expression of LINC00520 in GBM. Further experiments demonstrated that LINC00520 could interact with RNA-binding protein LIN28B to inhibit autophagy and reduce DNA damage, thereby contributing to TMZ chemoresistance in GBM. These findings suggested that STAT3/LINC00520/LIN28B axis might be a promising target to improve TMZ chemoresistance of GBM.

**Supplementary Information:**

The online version contains supplementary material available at 10.1186/s12935-022-02659-y.

## Background

Glioblastoma (GBM) is the most common primary malignant in the central nervous system [1, 2]. The major therapeutic strategies for GBM patients included surgical resection, adjuvant chemotherapy and radiotherapy [3, 4]. Due to high bioavailability across the blood brain barrier and few adverse effects, Temozolomide (TMZ) has been extensively used as the first-line chemotherapy regimen in GBM patients. It has been shown that GBM patients could benefit from TMZ treatment, with a survival improvement by 4.9 months [3]. However, a considerable number of GBM patients developed drug resistance to TMZ during chemotherapy, resulting in therapeutic failure and tumor recurrence. Various factors and underlying mechanism, including DNA damage repair, mismatch repair, the presence of cancer stem cells and autophagy, have been reported to be involved in TMZ chemoresistance [5–9]. However, the exact molecular mechanism is still not completely clarified.

As a group of RNA transcripts over 200 nucleotides in length, long noncoding RNAs (lncRNAs) are widely distributed in human genomics and play vital functions in multiple pathophysiological processes and human diseases [10, 11]. It has been reported that the dysregulation or dysfunction of numerous lncRNAs contributes to cell proliferation, invasion, migration and TMZ chemoresistance in GBM [12–15]. A recent study has demonstrated that LINC00520 regulated the tumorigenesis and metastasis of cutaneous squamous cell carcinoma by targeting epidermal growth factor receptor (EGFR) [16]. Also, EGFR signaling was identified as a key therapeutic target to overcome TMZ resistance [17, 18]. However, little known about the role of LINC00520 and its underlying mechanisms in TMZ chemoresistance of GBM. In terms of regulatory relationship, emerging evidence has suggested that the transcription of lncRNAs could be induced by transcription factors such as STAT3, C/EBPβ and SP1 [19–21]. As a transcription activator of lncRNAs, STAT3 function as an oncogene to promote malignant phenotype of GBM cells [22–24]. In addition, it has been shown that lncRNAs exert their oncogenic or anti-cancer role via acting as a miRNA sponge or interacting with RNA binding protein [25, 26]. In this study, bioinformatics analysis revealed a potential interaction between LINC00520 and Lin-28 Homolog B (LIN28B), a RNA-binding protein. Hence, we hypothesized that transcription factor STAT3-mediated upregulation of LINC00520 contributed to TMZ chemoresistance in GBM by interacting with LIN28B. This study might provide a novel insight into TMZ chemoresistance in GBM.

## Methods

All animal experiments were approved and conducted by the Animal Care and Ethics Committee of Lanzhou University Second Hospital.

### Cell lines and cell culture

The U251 and SKMG were two kinds of human GBM cell lines which were purchased from the Cell Bank of Chinese Academy of Sciences. The corresponding TMZ-resistant cell lines, which were named as U251/TMZ and SKMG-1/TMZ, were established by continuous selection with increasing concentrations of TMZ for 6 months. The cells were routinely cultured in DMEM medium (HyClone, USA) containing 10% fetal bovine serum (GIBCO, USA) and 100 U/ml streptomycin-penicillin (HyClone, USA) at a 37 °C humidified environment with 5% CO_2_. Furthermore, U251/TMZ and SKMG-1/TMZ cells were maintained in the medium with 50 µg/ml TMZ (Sigma, USA) to obtain a stable phenotype of TMZ resistance.

### Plasmid construction and cell transfection

The cDNA fragments with LINC00520 and/or LIN28B were synthesized and cloned into pcDNA3.1 vector (Invitrogen, USA). The short interfering RNAs (siRNA) for LINC00520 (si-LINC00520) and LIN28B (si-LIN28B) were designed to silence their expression in GBM cells (Shanghai, China), respectively. The transfection procedure was carried out following the manufacturer’s proposal. Briefly, GBM cells were inoculated into a six-well plate with a density of 5 × 10^5^ cells/well. Cells were then transfected by Lipofectamine 3000 (Invitrogen, USA) with pcDNA3.1 control vector, pcDNA3.1-LINC00520 vector, pcDNA3.1-LIN28B vector, siRNA control vector, si-LINC00520 vector and si-LIN28B vector, respectively. The efficiency of cell transfection was detected by qRT-PCR assay.

### RNA extraction and qRT-PCR

The extraction of total RNAs from TMZ-sensitive and TMZ-resistant GBM cells was performed using TRIzol Reagent (Invitrogen, USA). Then, 1 µg of extracted RNAs were used as a template to synthesize cDNA by means of a specific reverse transcription kit (Takara, China). The value of cycle threshold (Ct) was used to quantify the relative expression level of LINC00520, and the data was persented using.

the 2^−ΔΔ CT^ method.

### Cell counting kit (CCK-8)

The viability of GBM cells was measured through CCK-8 assay to investigate the effects of LINC00520 expression on TMZ chemoresistance. Briefly, the transfected TMZ-sensitive or TMZ-resistant cells were plated with 3000 cells per well. Each well was added 10 µL CCK-8 regent diluted with culture medium and was incubated for 2 h under 37 °C atmosphere with 5% CO_2_. The value of optical density under 490 nm was measured to detect the cell viability of GBM.

### Colony formation assay

For colony formation assay, TMZ-sensitive or TMZ-resistant GBM cells were planked with 5000 cells per well. After adherence growth, GBM cells were treated with 50 µg/ml TMZ for 24 h, and then were incubated with TMZ-free medium for 14 days. 4% formaldehyde and 0.1% crystal violet was used to fix and stain cell colonies, respectively.

### Flow cytometry analysis of cell apoptosis and cell cycle

After transfections, GBM cells and their TMZ-resistant cells were treated with 50 µg/ml TMZ for 48 h, and then were collected and digested with 0.25% trypsin. Next, these cells were resuspended by binding buffer at 4 °C and treated with 5 µL of FITC-labeled Annexin-V and propidium iodide (PI) reagent for 15 min under the dark. The apoptotic cells were immediately analyzed using flow cytometry (FACScan, USA). For the detection of cell cycle, GBM cells and their TMZ-resistant cells were additionally treated with serum starvation for 12 h to synchronize cell cycle. The cells were fixed with 70% ethanol at 4 °C overnight and stained with PI solution before flow cytometry of cell cycle.

### TUNEL assay

The apoptosis of GBM cells treated with TMZ was further measured by TUNEL assay. In briefly, GBM cells were harvested and incubated with 0.1% Triton X-100 (Solarbio, China). Then, GBM cells were fixed with 4% paraformaldehyde and apoptotic cells were labeled by the TUNEL reagent containing the rTdT enzyme (Roche, Germany). In addition, Nuclei were stained with DAPI. The amount of staining-positive cells was counted under six random fields using a fluorescent microscope (Nikon, Japan).

### Western blot

Radio-immunoprecipitation assay (RIPA) buffer with protease inhibitor was used to lyse GBM cells and extract total proteins, and their concentrations were quantified by bicinchonininc acid protein kit (Thermo, USA). The protein samples were electrophoretically separated by 10-12% SDS-PAGE gel and then were electrotransfered onto PVDF membranes (Millipore, USA). After sealing with 5% milk for 1 h, the membranes were incubated with primary antibodies against LIN28B (abcam, UK; ab229628, rabbit, polyclonal, 1:2500), LC3B (abcam, UK; ab48394, rabbit, polyclonal, 1:2000), Beclin-1 (abcam, UK; ab207612, rabbit, polyclonal, 1:2000) and β-actin (abcam, UK; ab8226, mouse monoclonal, 1:2000) under 4 °C overnight, respectively. Next, the membranes were incubated with the anti-rabbit or anti-mouse IgG HRP-labeled second antibody (1:2000) for 1 h at room temperature. The protein bands were generated by enhanced chemiluminescence (ECL) detection kit, and ECL detection system (GE Healthcare, Chicago, USA) was used for image observation.

### Chromatin immunoprecipitation (ChIP)

ChIP assay was carried out by a ChIP Assay Kit (Beyotime, China). In brief, U251, SKMG-1 and their TMZ-resistant cells were collected and fixed with 1% formaldehyde for 20 min under room temperature. DNA fragments with 200–500 bp were harvested using sonication lysis. The antibody against STAT3 (anti-STAT3, abcam, UK; ab119352, rabbit, monoclonal) was used to precipitate the chromatin and nonspecific antibody against IgG (anti-IgG, Thermo Fisher Scientific, USA) was used as a negative control. After that, the immunoprecipitated DNA were detected by qRT-PCR assays.

### Dual-luciferase reporter assay

The fragment sequence containing binding sites of STAT3 for the promoter regions of LINC00520 (Wild-type) were synthesized and cloned into luciferase reporter. The luciferase reporter with corresponding sites of mutant-type was used as a control. Then, U251, SKMG-1 and their TMZ-resistant cells were co-transfected with the luciferase reporters and STAT3-overexpressed vector or negative control vector. After transfection for 48 h, the luciferase activity was measured by Dual Luciferase Reporter Assay System (Promega, USA).

### RNA immunoprecipitation (RIP) assay

The RNA-Binding Protein Immunoprecipitation Kit (Millipore, USA) was used in RIP assay, and all procedures were performed in line with the manufacturer’s proposals. The antibody against LIN28B (abcam, UK; ab229628, rabbit, polyclonal) and anti-IgG antibody (Thermo Fisher Scientific, USA) were incubated with cell lysates at 4 °C overnight. The RNA-protein complexes were collected and the relative expression level of immunoprecipitated RNAs was analyzed by qPCR assay.

### Fluorescence in situ hybridization (FISH)

A total of five primary GBM and five recurrent GBM tissues were collected to detect the expression of LINC00520. All protocols were approved by the Ethics Review Board of our institution, and written informed consent for use of clinical sample was obtained from each subject. The FISH probe of LINC00520 was designed and synthesized by RiboBio (Guangzhou, China). In brief, the sections of fresh GBM tissues were fixed with 4% paraformaldehyde, washed by PBS for three times, and then were pretreated with prehybridization buffer at room temperature for 1 h. Next, the sections were hybridized using lncRNA FISH Probe Mix at 37 °C overnight. After hybridization, the expression of LINC00520 in primary and recurrent GBM tissues was detected using a fluorescent microscope (Olympus, Japan).

### Immunofluorescence and immunohistochemistry

Briefly, GBM cells were treated with TSA and then were fixed with 4% paraformaldehyde for 15 min, permeabilized by 0.5% Triton X-100 and blocked with 5% Bovine serum for 1 h at room temperature. Subsequently, cells were incubated with primary antibody against LIN28B (abcam, UK; ab229628, rabbit, polyclonal, 1:500) at 4 °C overnight. After washing with PBS for three times, the cells were incubated with fluorescent-labeled secondary antibody in the dark for 1 h. The nuclei were stained with DAPI, and the images were photographed under a fluorescent microscope (Olympus, Japan). Furthermore, the expression of LIN28B in primary and recurrent GBM tissues was detected by immunohistochemistry staining.

### Comet assay

GBM cells and their corresponding TMZ-resistant cells were treated with TMZ and then were subjected to comet assay to detect the DNA damage of these cells. All experiments were carried out according to the method described by previous studies [27, 28].

### Tumor xenograft model

Four-to-six-week-old male BALB/c nude mice were used to build orthotopic xenograft model in vivo. A total of 1 × 10^6^ U251/TMZ cells with stable LINC00520-silencing were injected into the right striatum of athymic BABL/c nude mice (n = 5). After 1 week, all nude mice in study group were treated with TMZ at the dose of 50 mg/kg for 5 days. The volume of tumor xenograft was measured each 7 days using Bioluminescence imaging system (IVIS Spectrum, USA).

### Statistical analysis

At least three replicates were done for all experiments, and the data were shown as mean ± standard deviation (SD). For continuous variables, Student’s t-test or one-way ANOVA test was used to compare the statistical difference between the groups if appropriate. The data processing and statistical analysis were carried out using SPSS 23.0 version software (IBM Corp, Chicago, USA). A p-value < 0.05 was regarded statistically significant.

## Results

### LINC00520 expression is increased in TMZ-resistant cells and its silencing improves TMZ chemoresistance

**Fig. 1:**
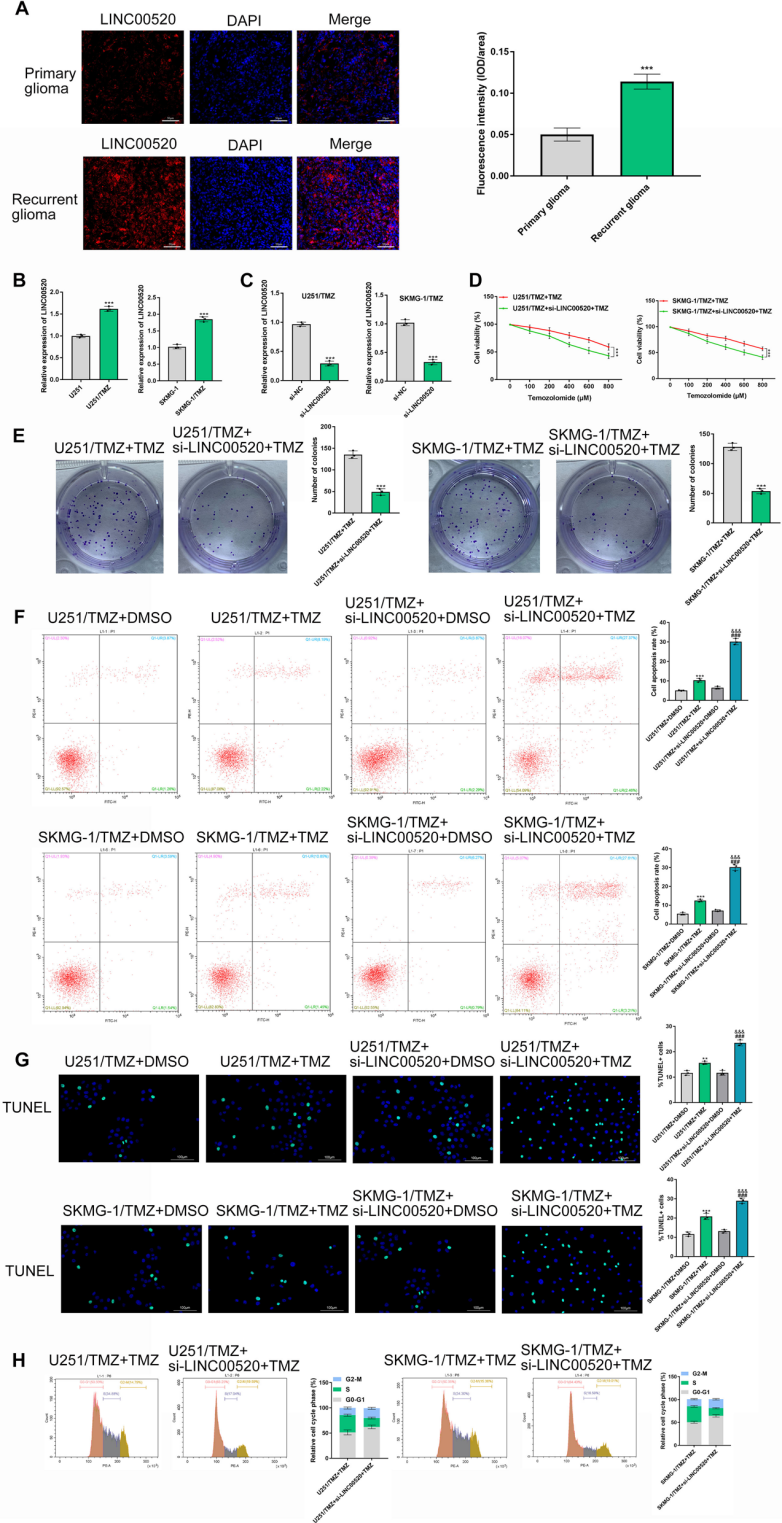
The expression of LINC00520 was increased in TMZ-resistant cells and its silencing improved TMZ chemoresistance in GBM. **A** The expression of LINC00520 in primary and recurrent GBM tissues was detected by FISH. **B** The expression levels of LINC00520 in TMZ-sensitive and TMZ-resistant cells were detected by qRT-PCR assay. **C** The efficiency of LINC00520 knockdown in U251/TMZ and SKMG-1/TMZ cells was detected after transfecting with siRNA for LINC00520. **D** LINC00520 knockdown decreased the cell viability of U251/TMZ and SKMG-1/TMZ under TMZ treatment (200 µM). **E** LINC00520 knockdown suppressed colony formation of U251/TMZ and SKMG-1/TMZ under TMZ treatment (200 µM). The effect of LINC00520 knockdown on cell apoptosis of U251/TMZ and SKMG-1/TMZ was investigated by flow cytometry (**F**) and TUNEL assay (**G**), respectively. **H** The distribution of cell cycle in U251/TMZ and SKMG-1/TMZ cells transfected with si-LINC00520 or si-NC were analyzed using flow cytometer

We firstly detected the expression levels of LINC00520 in primary and recurrent GBM specimens using FISH, and found that the expression of LINC00520 was significantly increased in recurrent GBM specimens (Fig. 1A). The expression levels of LINC00520 in TMZ-sensitive and TMZ-resistant GBM cells were then detected using qRT-PCR assay. We found that LINC00520 was significantly over-expressed in TMZ-resistant U251 cells in comparison to TMZ-sensitive U251 cells (P < 0.001), and similar finding was observed in SKMG-1 cell line and its TMZ-resistant counterpart (P < 0.001) (Fig. 1B).

To further explore the impact of LINC00520 on TMZ resistance of GBM in vitro, U251/TMZ and SKMG-1/TMZ cells were transfected with siRNA for LINC00520, and cells in the control group were transfected with siRNA empty vector (P < 0.001)(Fig. 1C). The CCK-8 assay demonstrated that the silencing of LINC00520 markedly reduced the cell viability of U251/TMZ and SKMG-1/TMZ under 200 µM TMZ treatment (P < 0.001) (Fig. 1D). Additionally, colony formation assays revealed that the amount of colonies in U251/TMZ and SKMG-1/TMZ cells were significantly decreased by LINC00520 silencing after TMZ treatment (P < 0.001) (Fig. 1E).

As a major kind of cell death induced by TMZ treatment, cell apoptosis analysis was further conducted by flow cytometry and TUNEL assay. After TMZ treatment for 48 h, it was observed that the silencing of LINC00520 resulted in an obvious raise in the amount of apoptotic cells (P < 0.001) (Fig. 1F). Likewise, the TUNEL assay revealed that the proportion of apoptotic cells was evidently higher in si-LINC00520 group than in control group (P < 0.001) (Fig. 1G). Cell cycle analysis showed that silencing of LINC00520 further reduced the percentage of S phase in U251/TMZ and SKMG-1/TMZ cells after TMZ treatment. These findings suggested that silencing of LINC00520 could reverse TMZ-resistant phenotype of GBM cells (Fig. 1H).

### Overexpression of LINC00520 contributed to TMZ chemoresistance in GBM cells

**Fig. 2:**
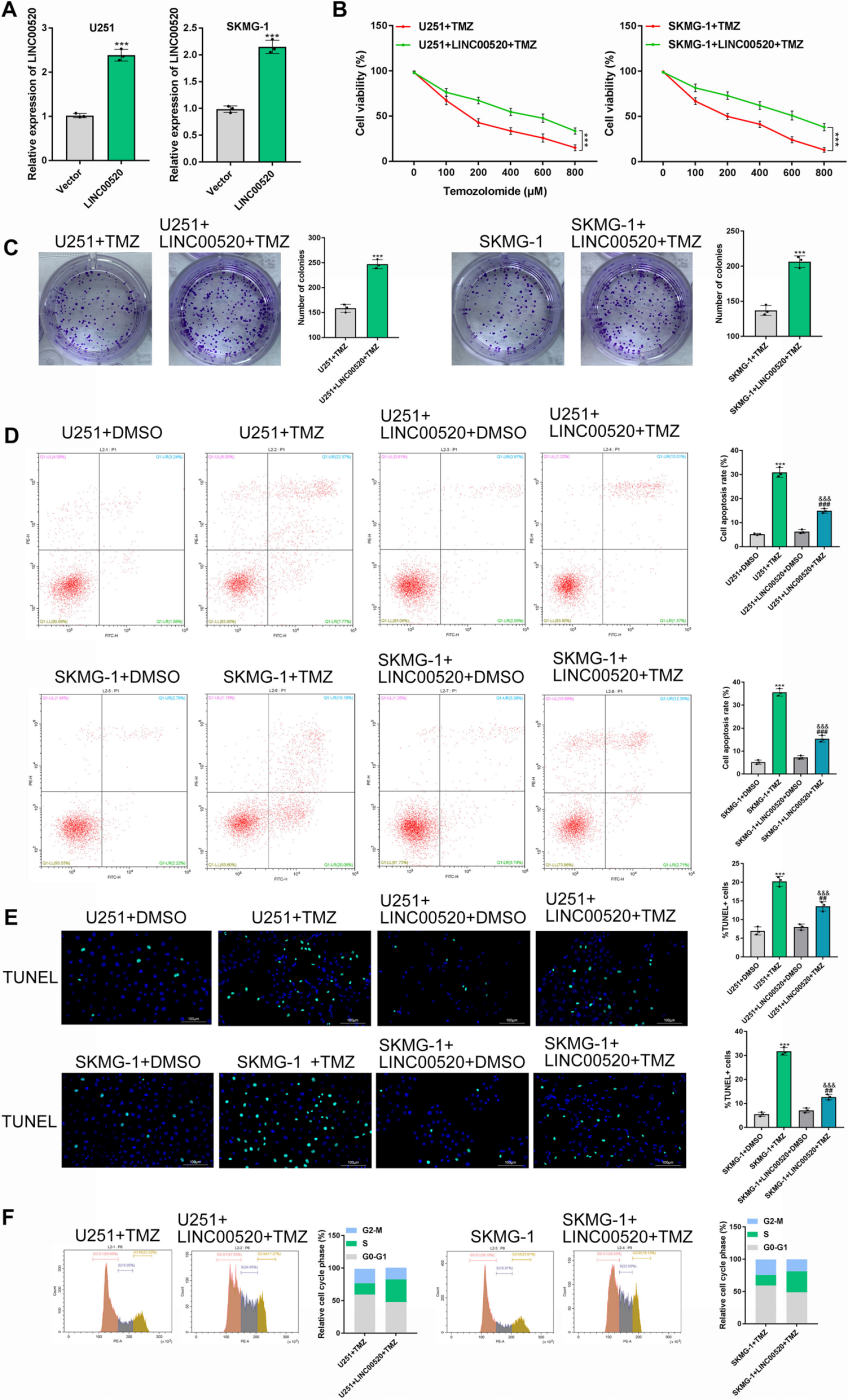
Overexpression of LINC00520 contributed to TMZ chemoresistance of GBM cells. **A** The efficiency of LINC00520 overexpression in U251 and SKMG-1 cells was detected by qRT-PCR analysis. **B** The viability of GBM cells treated with TMZ was significantly increased by LINC00520 overexpression. **C** LINC00520 overexpression promoted colony formation of U251 and SKMG-1 cells treated with 200 µM TMZ. The effect of LINC00520 overexpression on cell apoptosis of GBM was analyzed by flow cytometry (**D**) and TUNEL assay (**E**), respectively. **F** LINC00520 overexpression elevated the proportion of G1 phase in U251 and SKMG-1 cells treated with 200 µM TMZ

Based on the above findings, we also made a clear answer whether overexpression of LINC00520 contribute to TMZ chemoresistance in GBM cells. For this purpose, U251 and SKMG-1 cells with LINC00520 overexpression were constructed by pcDNA-LINC00520, and pcDNA3.1 empty vector was used as the negative control (P < 0.001) (Fig. 2A). CCK-8 assay and colony formation analysis demonstrated that the LINC00520 overexpression markedly elevated cell viability and accelerated the formation of GBM colonies under TMZ treatment (P < 0.001) (Fig. 2BC). In contrast, the proportion of apoptotic cells in U251 and SKMG-1 was significantly decreased by the overexpression of LINC00520 using flow cytometry analysis (P < 0.001) (Fig. 2D). Also, the TUNEL assay was carried out in U251 and SKMG-1 cells treated with 50 µg/ml TMZ, and a lower rate of cell apoptosis was observed in LINC00520 overexpression group than in the control group (P < 0.001) (Fig. 2E). The S phase proportion of U251 and SKMG-1 cells were decreased by TMZ treatment, but the overexpression of LINC00520 elevated the S phase proportion of these cells (Fig. 2F). Therefore, we concluded that the overexpression of LINC00520 induced chemotherapy resistance to TMZ in GBM cells.

### STAT3 promoted the transcription of LINC00520 via binding to its promoter

**Fig. 3:**
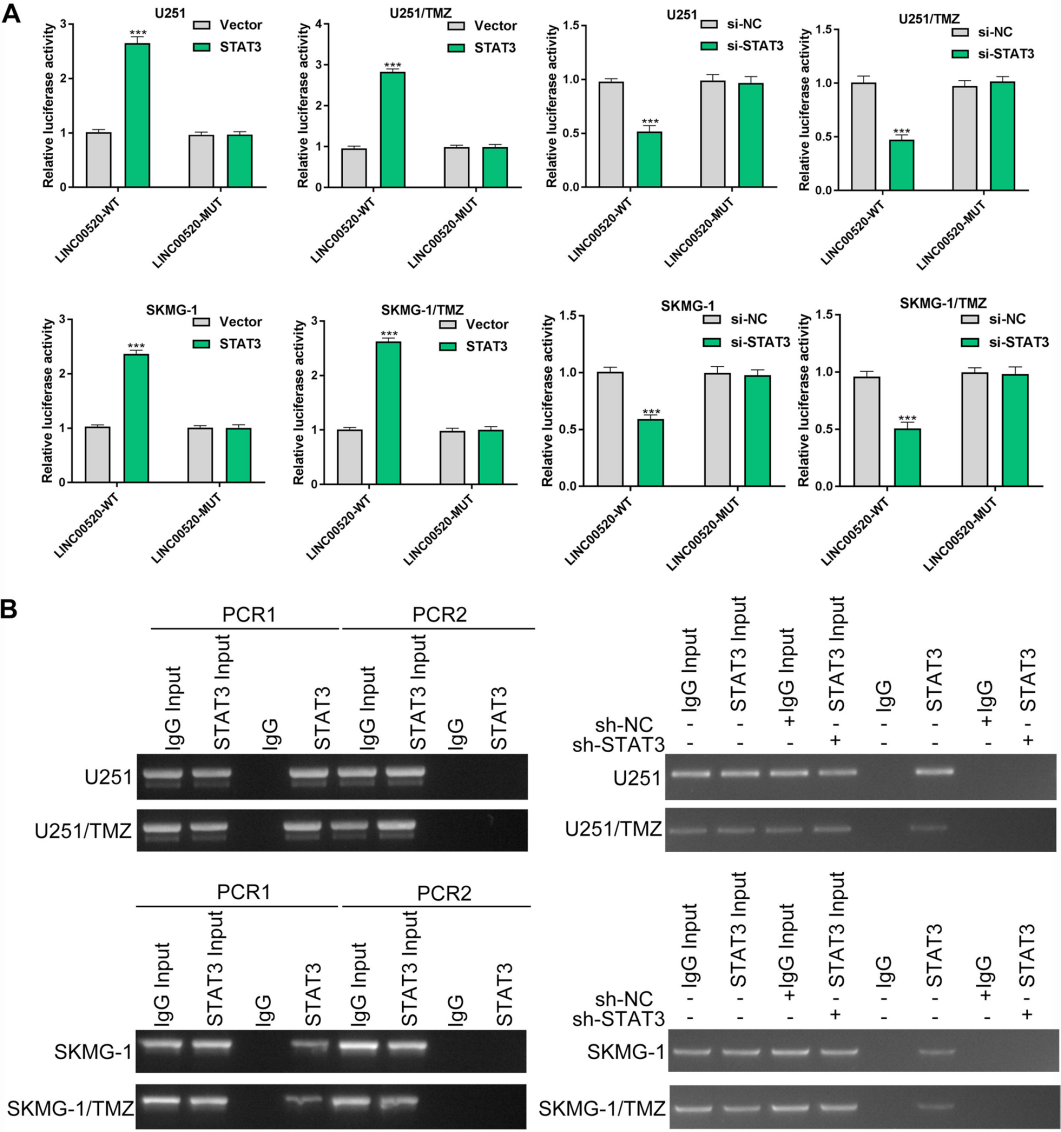
STAT3 promoted the transcription of LINC00520 via binding to its promoter. **A** Luciferase reporter assays revealed that LINC00520 expression in TMZ-sensitive and TMZ-resistant cells could be regulated by transcription factor STAT3. **B** ChIP assay further supported that STAT3 was a key regulator of LINC00520 transcription (PCR 1 was conducted using the specific primer for LINC00520, and PCR 2 was conducted using the control primer)

The transcription factors might be involved in the dysregulation of lncRNAs expression in human cancers. In this study, an online prediction tool, JASPAR, was used to explore the potential transcription factors at the upstream of LINC00520. The results revealed that STAT3 had potential binding sites at the promoter regions of LINC00520. The qRT-PCR assay showed that TMZ-resistant cells had a higher level of STAT3 expression than parental GBM cells (Additional file 1: Fig. S1). Next, the luciferase reporter assay was performed to determine the interaction between transcription factor STAT3 and the promoter regions of LINC00520. It was found that overexpression of STAT3 significantly elevated luciferase activity of wild-type LINC00520 in TMZ-sensitive and TMZ-resistant cells, while did not alter the luciferase activity of mutant-type LINC00520 (P < 0.001) (Fig. 3A). Similar findings were observed in GBM cells transfected with siRNA for LINC00520 (P < 0.001) (Fig. 3A). More importantly, the ChIP assay further demonstrated that STAT3 could specifically bind to the promoter regions of LINC00520 (Fig. 3B). These findings suggested that STAT3 activated the transcription of LINC00520 via binding to its promoter.

### LINC00520 contributed to TMZ chemoresistance in GBM cells by interacting with LIN28B

**Fig. 4:**
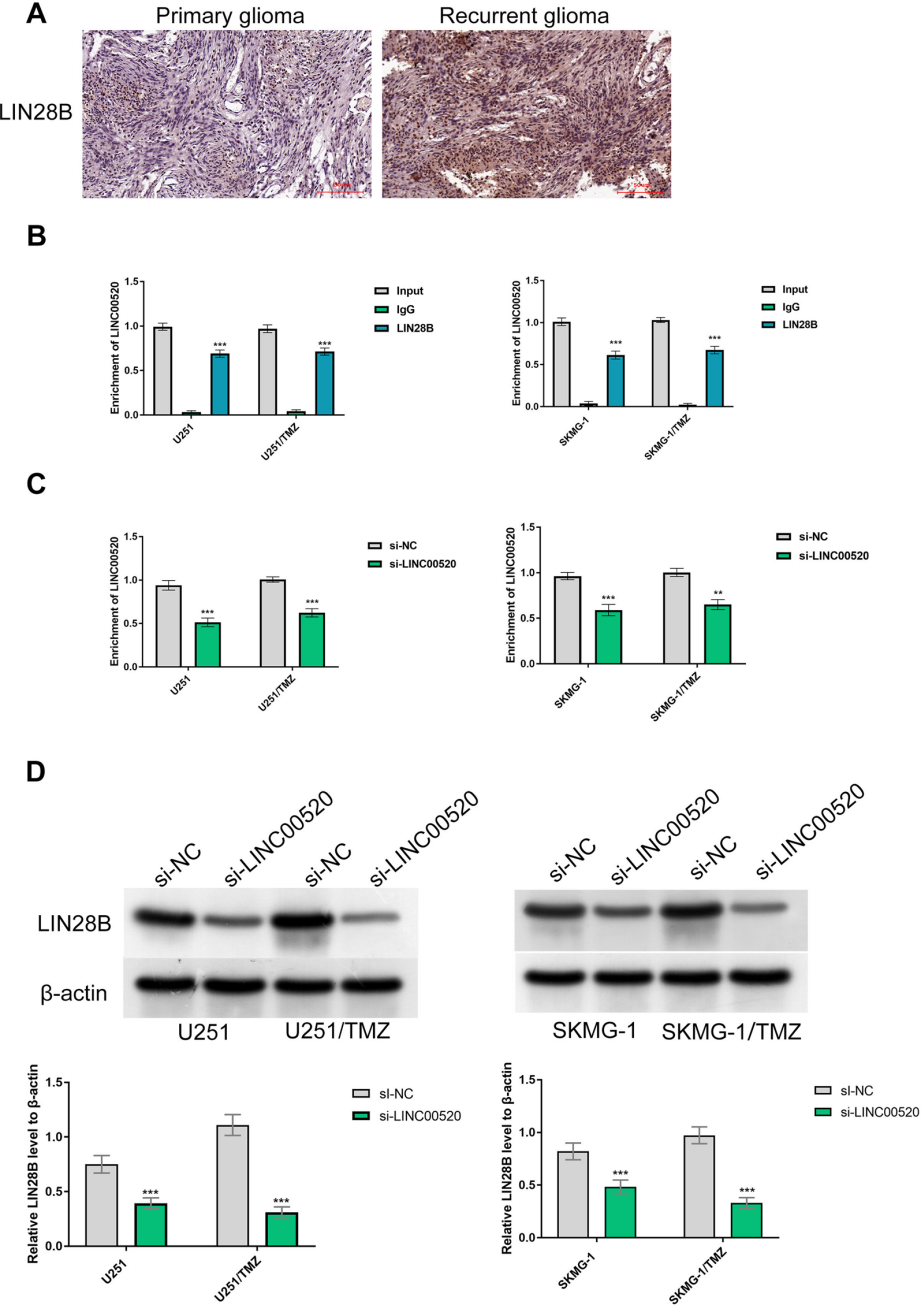
LINC00520 contributed to TMZ chemoresistance by interacting with LIN28B. **A** LIN28B was overexpressed in recurrent GBM samples compared to primary GBM samples. **B** RNA immunoprecipitation assay revealed that LINC00520 was markedly enriched in both TMZ-sensitive and TMZ-resistant cells using anti-LIN28B antibody, suggesting an interaction between LINC00520 and LIN28B. qRT-PCR (**C**) and Western blot assay (**D**) consistently demonstrated that the expression of LIN28B was positively related to LINC00520 expression in GBM cells

We used the catRAPID algorithm to screen protein complexes that had a potential interaction to LINC00520. As an RNA-binding protein, LIN28B was identified as a target that binds to LINC00520. Similar to the expression of LINC00520, LIN28B was observed to be overexpressed in recurrent GBM samples compared to primary GBM samples (Fig. 4A). We further carried out a RNA immunoprecipitation assay to validate the interaction between LINC00520 and LIN28B in GBM cells and their TMZ-resistant counterparts. Using a specific antibody against LIN28B, we observed that LINC00520 was significantly enriched in these cells (P < 0.001) (Fig. 4B). qRT-PCR and Western blot assay consistently indicated that the expression levels of LIN28B in TMZ-sensitive and TMZ-resistant cells were markedly decreased by the silencing of LINC00520 (P < 0.001) (Fig. 4C and D). These data supported a positive relationship between the expression of LINC00520 and LIN28B, and LINC00520 could directly interact with LIN28B.

### LIN28B contributed to TMZ chemoresistance by inhibiting cell autophagy and reducing DNA-damage response

**Fig. 5:**
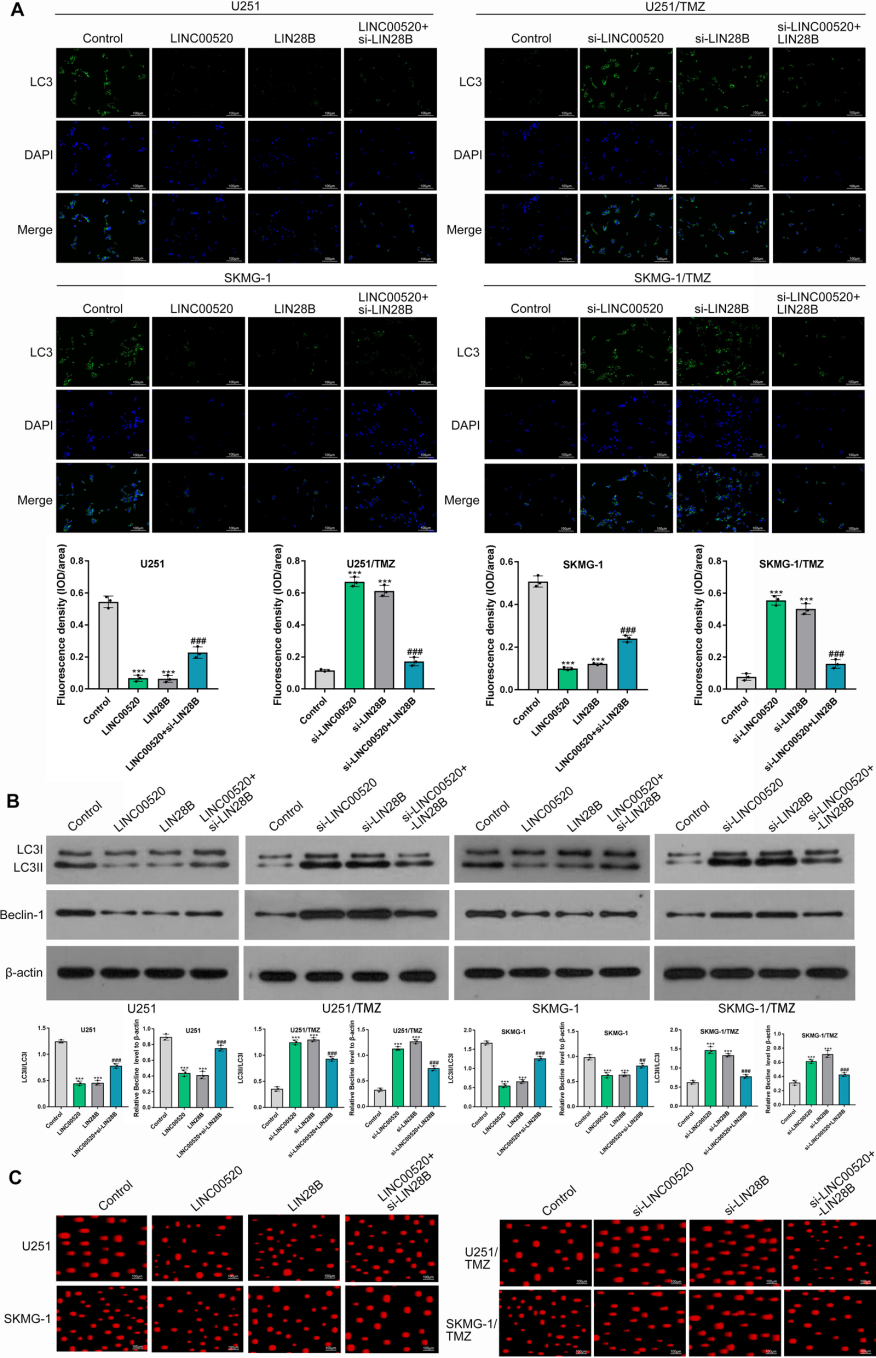
LIN28B contributed to TMZ chemoresistance by inhibiting cell autophagy and reducing DNA-damage response. **A** The effects of LINC00520/LIN28B axis on cell autophagy of GBM were measured by immunofluorescence labeled with GFP-LC3. **B** The expression of autophagy-related proteins, including LC3II, LC3I and Beclin-1, were detected by Western blot. **C** The alkaline comet assay indicated that LINC00520/LIN28B axis contributed to TMZ chemoresistance in GBM via reducing DNA damage response

To further determine the underlying mechanism of TMZ chemoresistance mediated by LIN28B, we explored the impact of LIN28B on cell autophagy in GBM. As shown in Fig. 5A, immunofluorescence staining with green fluorescent protein (GFP) labeled LC3 showed that overexpression of LINC00520 or LIN28B had a lower proportion of GFP-LC3 puncta than the negative control, while this trend could be partially reversed by LIN28B knockdown (Fig. 5A). Consistently, Western blot analysis suggested that the expression levels of autophagy-related proteins, particularly, LC3II/LC3I protein ratio and Beclin-1, were evidently reduced by overexpression of LINC00520 or LIN28B (P < 0.001) (Fig. 5B). However, LIN28B knockdown partially restored the LC3II/LC3I protein ratio and the expression of Beclin-1in GBM cells. Moreover, the silencing of LINC00520 or LIN28B evidently increased the expression of autophagy-related proteins in TMZ-resistant cells, while this effect could be partially reversed by upregulation of LIN28B (P < 0.001) (Fig. 5B). These data suggested that LIN28B might mediate the promoting effect of LINC00520 on TMZ chemoresistance via reducing autophagy of GBM cells.

We also investigated the impact of LINC00520/LIN28B axis on DNA damage response of GBM cells after TMZ treatment. As shown in Fig. 5C, the proportion of DNA in the tail was significantly decreased by upregulation of LINC00520 or LIN28B, while the DNA damage response was induced by LIN28B knockdown again. In contrast, the silencing of LINC00520 or LIN28B markedly increased DNA damage of TMZ-resistant U251 and SKMG-1 cells (Fig. 5C). These findings provided another explanation that LINC00520/LIN28B axis might contribute to TMZ chemoresistance in GBM by decreasing drug-induced DNA damage.

### Silencing of LINC00520 improved the response to TMZ chemotherapy in vivo

**Fig. 6:**
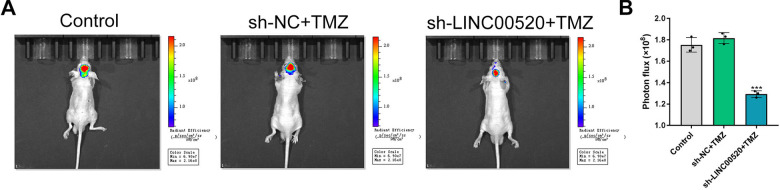
Silencing of LINC00520 improved the response to TMZ chemotherapy in vivo. **A** Representative images of intracranial xenografts originated from U251/TMZ cells transfected with si-LINC00520 vector or si-NC vector under TMZ treatment. **B** The volume of tumor xenografts were evidently reduced by transfecting with si-LINC00520, suggesting that silencing of LINC00520 improve TMZ chemoresistance of GBM cells in vivo

The orthotopic GBM xenograft model was established to investigate the effect of LINC00520 on response to TMZ chemotherapy in vivo. After injection of TMZ-resistant U251 cells transfected with si-LINC00520 vector or si-NC vector, all nude mice in two groups were treated with TMZ 5 days per week for three cycles (Fig. 6A and Additional file 1: Fig. S1). Using bioluminescence imaging system, we found that the volume of intracranial tumor xenografts were markedly reduced by the silencing of LINC00520, while a significant progression in tumor growth was observed in the si-NC group (P < 0.001) (Fig. 6B). These findings demonstrated that silencing of LINC00520 could reverse TMZ chemoresistance of GBM cells in vivo.

## Discussion

It is difficult to achieve complete surgical resection due to aggressive biological behaviors, making adjuvant TMZ treatment indispensable for GBM patients. Emerging evidences have suggested that lncRNAs are involved in the development of TMZ resistance in GBM cells. A recent report has shown that lncRNA SNHG12 is aberrantly expressed in GBM samples and it induces GBM chemoresistance to TMZ by facilitating the cell growth and suppressing cell apoptosis [15]. More recently, lncRNA SOX2OT has been shown to be a prognostic marker for GBM patients and confer chemoresistance to TMZ by regulating SOX2 expression, thereby driving Wnt5a/β-catenin pathway [29]. In the present study, our data suggested that the expression of LINC00520 was obviously upregulated in recurrent GBM samples and TMZ-resistant cells. The knockdown of LINC00520 reversed TMZ resistance of GBM cells via suppressing cell proliferation, inducing cell apoptosis and G1/S phase arrest. In contrast, overexpression of LINC00520 facilitated to TMZ chemoresistance in GBM by accelerating cell growth and suppressing cell apoptosis.

As a novel identified lncRNA, LINC00520 was reported to be located on chromosome 14 and markedly overexpressed in human cancers such as malignant melanoma, colorectal carcinoma and non-small cell lung cancer (NSCLC) [30–32]. A recent study reported a high expression of LINC00520 in glioma samples and cell lines. Functionally, LINC00520 accelerated cell growth, invasion, migration and reduce cell apoptosis of glioma [33]. Similarly, the study of Jin et al. reported that LINC00520 function as a tumor promoter in development and metastasis of colorectal carcinoma via sponging miR-577 and thereby regulating the expression of heat shock protein 27 (HSP27) [32]. The effect of LINC00520 on biological behaviors of cancer cells promoted us to further investigate its roles in GBM chemoresistance. Our results revealed that LINC00520 might act as a potential target to overcome TMZ chemoresistance in GBM.

Subsequently, we explored the underlying mechanism of dysregulation of LINC00520 expression in TMZ-resistant GBM cells. Accumulating evidences have suggested that aberrant expression of lncRNAs could be regulated by transcription factors such as C/EBPβ, USF1 and SP1 [20, 21, 34]. Using JASPAR database, STAT3 was identified as a potential regulator at the upstream of LINC00520. More importantly, both luciferase reporter assay and ChIP assay revealed a strong affinity of STAT3 to the promoter regions of LINC00520, suggesting the overexpression of LINC00520 in GBM cells might be mediated by transcription factor STAT3. As a well-known oncogenic transcription factor, STAT3 has been shown to activate the transcription of a few functional lncRNAs in human cancers [35–37]. Pan et al. found that STAT3 induced overexpression of lncRNA SNHG17 and thus played an oncogenic role in pathogenesis and progression of ovarian cancer [36]. Similarly, the study of Whitney et al. demonstrated that the transcription factor STAT3 was crucially essential for the regulation of LINC00520 expression, which contributed to malignant phenotype of breast cancer cells in vitro [37]. In this study, our results showed that transcription factor STAT3 act as a key regulator of LINC00520 in GBM chemoresistance to TMZ. These findings further strengthened the regulatory role of transcription factor STAT3 in the transcription of lncRNA.

In addition to being a miRNA sponge, lncRNAs could bind to target proteins to regulate gene expression at the transcriptional and posttranscriptional level. For example, He et al. reported that lncRNA MIR155HG could contribute to TMZ chemoresistance in GBM cells by interacting with RNA binding protein PTBP1 [38]. Another example was that LIN28B-AS1 accelerated the proliferation and metastasis of lung adenocarcinoma cells by binding to IGF2BP1 protein [39]. In the present study, we used the catRAPID algorithm to identify an RNA-binding protein, LIN28B, as a target to LINC00520. Our experiments further validated the interaction between LINC00520 and LIN28B in TMZ-sensitive and TMZ-resistant cells, implying that LINC00520/LIN28B axis might be involved in aggressive biological behaviors and TMZ-resistant phenotype of GBM. In fact, previous reports have shown that LIN28B is evidently overexpressed and plays a tumor-promoter role in multiple human cancers [40–42]. The study of Wang et al. reported that LIN28B function as an oncogenic “driver” to promote proliferation and metastasis of lung adenocarcinoma cells in vitro and tumorigenicity in vivo [39]. Furthermore, the research on the mechanism of malignant phenotype mediated by LIN28B revealed that LIN28B could regulate cell cycle and affect DNA damage repair [39].

These observations draw our attention to further explore the underlying mechanism of TMZ chemoresistance mediated by LIN28B. Autophagy has been shown to be implicated in the viability of cancer cells and considered to be a key cause of chemotherapy resistance [9, 43]. In this study, we proved that LINC00520/LIN28B axis might suppress cell autophagy in GBM, thus contributing to TMZ-resistant phenotype. By contrast, knockdown of LINC00520 and/or LIN28B reversed TMZ chemoresistance of GBM cells by activating autophagy. However, the exact mechanism behind the role of autophagy in TMZ chemoresistance need be further investigated in the future. Also, chemotherapy drugs-mediated DNA double-strand break is repaired by damage repair response, which is considered as another vital mechanism of TMZ chemoresistance in GBM [5, 44]. Our data suggested that DNA damage induced by TMZ treatment could be inhibited by LINC00520/LIN28B axis, while its silencing lead to an obvious raise in DNA damage of GBM cells. These findings might provide a novel way for overcoming TMZ chemoresistance in GBM.

## Conclusions

To summarize, our results demonstrated that transcription factor STAT3 mediated dysregulation of LINC00520 in GBM, and overexpression of LINC00520 contributed to TMZ chemoresistance by accelerating cell proliferation and reducing cell apoptosis. In terms of molecular mechanism, LINC00520 could interact with RNA-binding protein LIN28B to inhibit autophagy and reduce DNA damage, thereby conferring TMZ-resistant phenotype of GBM cells. These findings suggested that STAT3/LINC00520/LIN28B axis might be a promising target to improve TMZ chemoresistance of GBM.

## Supplementary Information

**Additional file 1: Figure S1.** (A). The expression levels of STAT3 in parental GBM cells and TMZ-resistant cells were detected by qRT-PCR assay. (B). The expression levels of LINC00520 and LIN28B in tumor xenografts were detected by qRT-PCR assay.

## Data Availability

All data generated or analyzed during this study are included in this published article.
