# Supplementary figures and images for: STAT3-mediated upregulation of LINC00520 contributed to temozolomide chemoresistance in glioblastoma by interacting with RNA-binding protein LIN28B
Source: Cancer Cell Int. 2022 Aug 9;22:248. doi: 10.1186/s12935-022-02659-y (PMC9361558; doi:10.1186/s12935-022-02659-y)

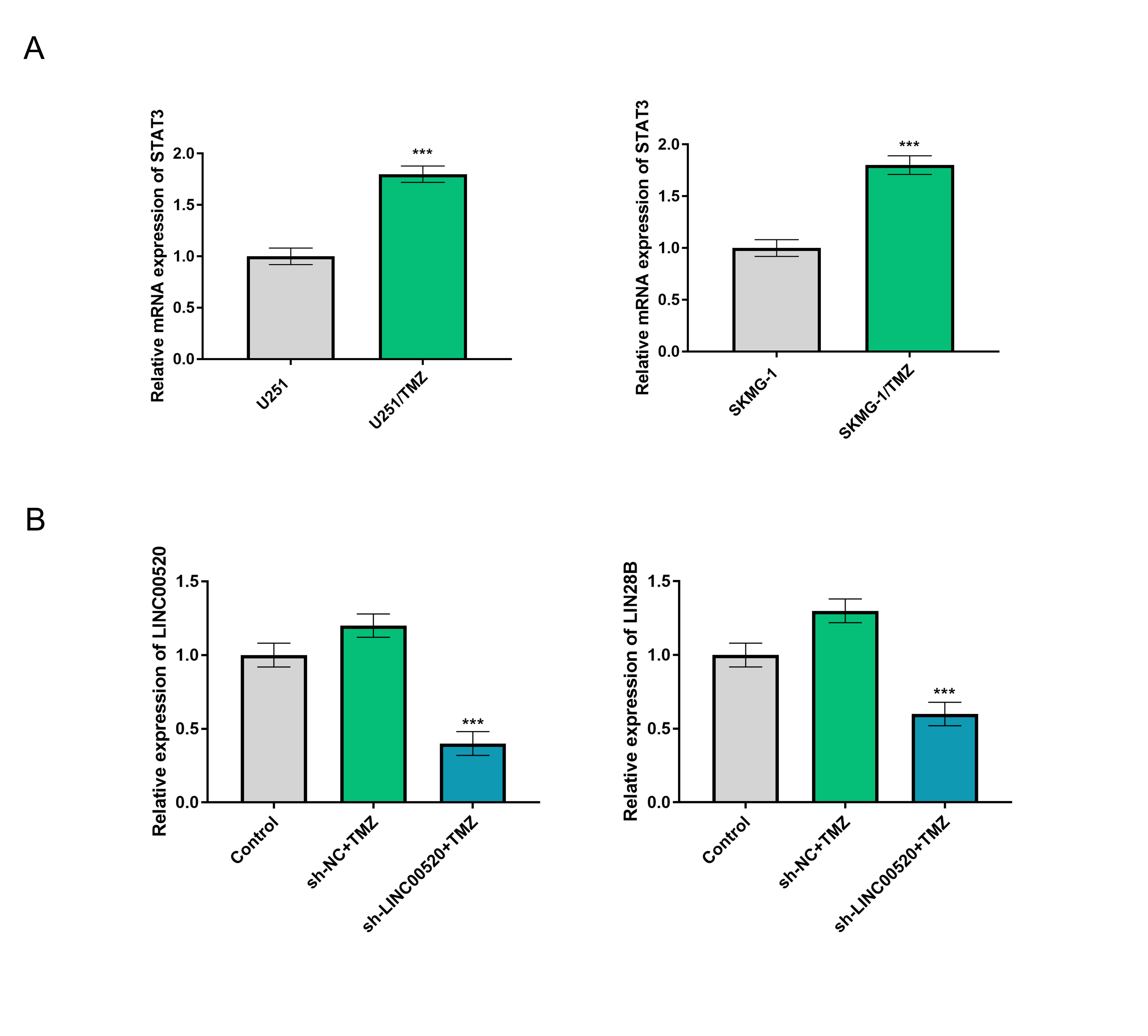

Supplement: Supplementary file 1 — Additional file 1: Figure S1. (A). The expression levels of STAT3 in parental GBM cells and TMZ-resistant cells were detected by qRT-PCR assay. (B). The expression levels of LINC00520 and LIN28B in tumor xenografts were detected by qRT-PCR assay. [file 12935_2022_2659_MOESM1_ESM.jpg]
